# Supplementary figures and images for: Enhancing sludge dewaterability in sequential bioleaching: Degradation of dissolved organic matter (DOM) by filamentous fungus Mucor sp. ZG-3 and the influence of energy source
Source: PLoS One. 2024 May 30;19(5):e0302311. doi: 10.1371/journal.pone.0302311 (PMC11139263; doi:10.1371/journal.pone.0302311)

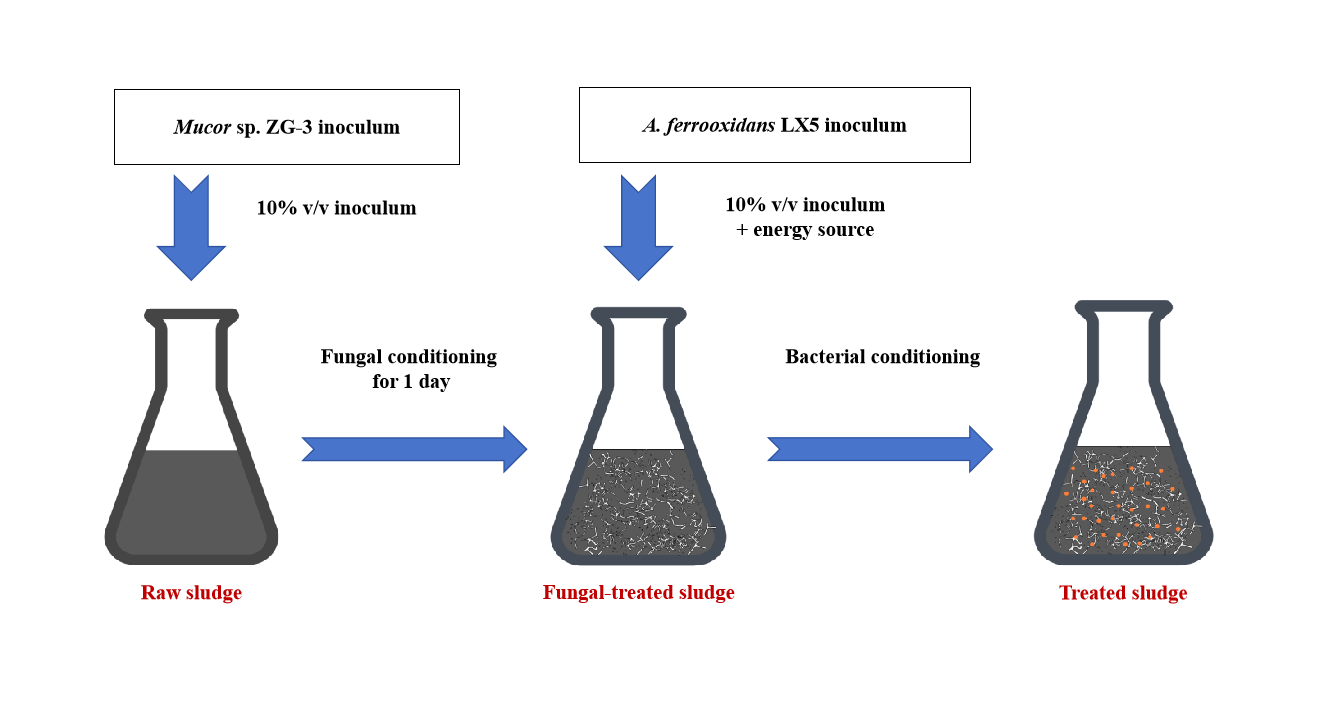

Supplement: S1 Fig — (DOCX) [file pone.0302311.s001.docx]
